# Surveillance for *Ixodes scapularis* and *Ixodes pacificus* ticks and their associated pathogens in Canada, 2020

**DOI:** 10.14745/ccdr.v49i06a06

**Published:** 2023-06-01

**Authors:** Christy Wilson, Salima Gasmi, Annie-Claude Bourgeois, Jacqueline Badcock, Justin Carr, Navdeep Chahil, Heather Coatsworth, Antonia Dibernardo, Priya Goundar, Patrick Leighton, Min-Kuang Lee, Muhammad Morshed, Marion Ripoche, Jade Savage, on behalf of eTick, Hanan Smadi, Christa Smolarchuk, Karine Thivierge, Jules Koffi

**Affiliations:** 1 Centre for Food-borne, Environmental and Zoonotic Infectious Diseases, Public Health Agency of Canada, Ottawa, ON; 2 Centre for Food-borne, Environmental and Zoonotic Infectious Diseases, Public Health Agency of Canada, Saint-Hyacinthe, QC; 3 Public Health New Brunswick, New Brunswick Department of Health, Fredericton, NB; 4 New Brunswick Provincial Veterinary Laboratory, Department of Agriculture, Aquaculture and Fisheries, Fredericton, NB; 5 BCCDC Public Health Laboratory, BC Centre for Disease Control, Vancouver, BC; 6 National Microbiology Laboratory Branch, Public Health Agency of Canada, Winnipeg, MB; 7 Ministry of Health, Regina, SK; 8 Epidemiology of Zoonoses and Public Health Research Group (GREZOSP), Faculty of Veterinary Medicine, Université de Montréal, Saint-Hyacinthe, QC; 9 Department of Pathology and Laboratory Medicine, University of British Columbia, Vancouver, BC; 10 Institut national de santé publique du Québec, Montréal, QC; 11 Bishop’s University, Sherbrooke, QC; 12 Analytics and Performance Reporting Branch, Health Standards, Quality and Performance Division, Alberta Health, Edmonton, AB; 13 Laboratoire de santé publique du Québec, Sainte-Anne-de- Bellevue, QC; 14 Institute of Parasitology, McGill University, Sainte-Anne-de- Bellevue, QC

**Keywords:** *Ixodes scapularis*, *Ixodes pacificus*, surveillance, *Borrelia*, *Anaplasma*, *Babesia*, Powassan virus

## Abstract

**Background:**

*Ixodes scapularis* and *Ixodes pacificus* ticks are the principal vectors of the agent of Lyme disease and several other tick-borne diseases in Canada. Tick surveillance data can be used to identify local tick-borne disease risk areas and direct public health interventions. The objective of this article is to describe the seasonal and spatial characteristics of the main Lyme disease vectors in Canada, and the tick-borne pathogens they carry, using passive and active surveillance data from 2020.

**Methods:**

Passive and active surveillance data were compiled from the National Microbiology Laboratory Branch (Public Health Agency of Canada), provincial and local public health authorities, and eTick (an online, image-based platform). Seasonal and spatial analyses of ticks and their associated pathogens are presented, including infection prevalence estimates.

**Results:**

In passive surveillance, *I. scapularis* (n=7,534) were submitted from all provinces except Manitoba and British Columbia, while *I. pacificus* (n=718) were submitted only from British Columbia. No ticks were submitted from the Territories. The seasonal distribution of *I. scapularis* submissions was bimodal, but unimodal for *I. pacificus*. Four tick-borne pathogens were identified in *I. scapularis* (*Borrelia burgdorferi*, *Anaplasma phagocytophilum*, *Babesia microti* and *Borrelia miyamotoi*) and one in *I. pacificus* (*B. miyamotoi*). In active surveillance, *I. scapularis* (n=688) were collected in Ontario, Québec and New Brunswick. Five tick-borne pathogens were identified: *B. burgdorferi*, *A. phagocytophilum*, *B. microti, B. miyamotoi* and Powassan virus.

**Conclusion:**

This article provides a snapshot of the distribution of *I. scapularis* and *I. pacificus* and their associated human pathogens in Canada in 2020, which can help assess the risk of exposure to tick-borne pathogens in different provinces.

## Introduction

*Ixodes scapularis* and *Ixodes pacificus* ticks can transmit several bacterial, viral and protozoan pathogens to humans ((1)). The geographic range and population of *I. scapularis* is increasing in southern central and eastern Canada ((2,3)), due to climate and environmental changes that have enhanced habitat suitability for ticks in more areas ((4,5)). These changes can further alter tick behaviour and extend their periods of activity, which can increase exposure to tick-borne diseases (TBD) ((1,6)). To reduce the burden from TBD, the continued range expansion of ticks in Canada must be met with increased capacity for and awareness of TBD prevention and surveillance ((1)). Tick surveillance data inform the environmental risk of Lyme disease (LD), which can guide public health authorities in targeting prevention and control efforts and support LD diagnostics by healthcare professionals ((7)).

The causative agent of LD, *Borrelia burgdorferi*, is transmitted by *I. scapularis* in central and eastern Canada and by *I. pacificus* in British Columbia. Reported incidence of LD in people has increased more than 10-fold (from 144 to 1,615 cases) from 2009 to 2020 ((8)). Additional TBD, transmitted by *I. scapularis* or *I. pacificus,* are emerging in Canada; including anaplasmosis ((9)), babesiosis ((10)), hard tick-borne relapsing fever ((11)) and Powassan virus disease ((12)).

Passive tick surveillance has been used since the 1990s to identify *I. scapularis* and *I. pacificus* tick populations and the presence of tick-borne pathogens ((13,14)). Active tick surveillance began in the 2000s to detect areas with established tick populations where LD risk may become endemic (LD risk areas) ((15)). Efforts to summarize passive and active tick surveillance annually at the national level began in 2019 ((16)), providing a baseline for TBD risk that over time will facilitate the identification of current trends and enable the projection of future trends.

The objective of this surveillance report is to summarize the geographic and seasonal characteristics of the main LD vectors in Canada, *I. scapularis* and *I. pacificus*, collected through passive and active surveillance in 2020. This article will also summarize the prevalence and spatial distribution of their associated human pathogens.

## Methods

### Data sources

This report uses two types of surveillance data from ten different providers. Passive tick surveillance data was provided by the National Microbiology Laboratory (NML) Branch of the Public Health Agency of Canada (PHAC), British Columbia Centre for Disease Control (BCCDC), Alberta Health, Saskatchewan Ministry of Health, and eTick. Active tick surveillance data were provided by Thunder Bay District Health Unit, Kingston, Frontenac and Lennox & Addington Public Health, *Laboratoire de santé publique du Québec*, New Brunswick Department of Health and New Brunswick Provincial Veterinary Laboratory.

### Passive tick surveillance

Passive tick surveillance is the voluntary submission by the public of ticks (or their images) to medical or veterinary clinics, regional public health authorities or other institutions (e.g. university laboratory) for species identification and laboratory testing ((13)). This analysis was limited to *I. scapularis* and *I. pacificus* ticks collected within Canada in 2020, although several other tick species were also identified. Ticks could be submitted at any point during the year. Ticks with a location of acquisition outside of Canada, with a submitter's history of travel to another province, or from within Canada but could not be geocoded were excluded. Ticks were submitted individually (single submission) or in groups of two or more (multiple submission). Provinces with five or fewer ticks submitted for species identification and laboratory testing were excluded from the study to avoid misinterpretation of results. No ticks were submitted from Northwest Territories, Nunavut or Yukon as no passive surveillance programs exist for *I. scapularis* and *I. pacificus*.

Since 2009, regional passive tick surveillance programs have been gradually discontinued in several jurisdictions (e.g. Nova Scotia, southwestern Québec and eastern Ontario) dependent on laboratory capacity and as *I. scapularis* populations have become established. However, ticks (or their images) acquired in these jurisdictions could be submitted by the public directly to NML or to eTick.

eTick is a validated, web-based, community-science passive surveillance system for tick identification ((17)). Individuals submit images of ticks they encounter to the online platform, which are then examined by trained personnel for species identification. The system began in 2017 in Québec, with five additional provinces added by 2020 (Saskatchewan, Ontario, Newfoundland and Labrador, New Brunswick and Nova Scotia). Similar to provincial tick surveillance data sources, eTick collects information on location of acquisition, date of collection, submitter travel history, tick host, tick species and tick instar. All ticks from eTick were classified as single submissions, as users must upload images of each tick individually.

Ticks acquired and submitted in Saskatchewan, Ontario, Québec, Newfoundland and Labrador, New Brunswick, Nova Scotia and Prince Edward Island were tested for *A. phagocytophilum*, *B. burgdorferi*, *B. miyamotoi* and *B. microti* at NML or University of Saskatchewan using the methods previously described ((16,18)). Ticks from BCCDC were tested only for *B. burgdorferi* and *B. miyamotoi* ((14)). Laboratory results for ticks from Alberta Health were not available. Specimens from tick records submitted through eTick were not routinely requested for testing of tick-borne pathogens but could be forwarded onto a laboratory for this purpose at the request of local public health authorities.

### Active tick surveillance

In active surveillance, ticks are collected from the environment using drag sampling or by capturing host mammals that are then examined for ticks. This analysis used *I. scapularis* ticks collected during drag sampling from 7 sites in Ontario, 24 sites in Québec and 14 sites in New Brunswick. Drag sampling takes place in late spring/summer (May through July) and fall (September through November), with some sites visited during both periods.

All ticks were tested at NML for *A. phagocytophilum*, *B. microti*, *B. burgdorferi*, *B. miyamotoi* and Powassan virus. Ticks were collected and tested using the methods previously described ((16,18,19)).

## Analysis

### Tick characteristics

For passive surveillance, descriptive statistics were calculated for submission type (sample-based or image-based), tick species, province of acquisition, instar (larva, nymph, adult female or adult male), level of engorgement (unfed or engorged), host (human, dog, cat or other) and month of collection. Where date of collection was not available, the date the sample was received was used to ascertain the month of collection. For active surveillance, descriptive statistics were calculated for province of collection and instar (larva, nymph, adult female or adult male). All data were cleaned and analysed in R (version 4.0.2).

Ticks that were acquired in Canada in passive surveillance were mapped using QGIS (version 3.8.1) based on their location of acquisition, except for ticks from Alberta that were mapped to the centroid of the forward sortation area (the first three characters of the postal code) of acquisition. Ticks from submitters with a history of travel in the previous 14 days within the same province as the locality of acquisition were geocoded to the location of exposure during travel. Ticks from submitters with multiple travel locations listed were not mapped. In active surveillance, the location of tick dragging was geocoded and mapped.

### Infection prevalence

To account for pooled testing of ticks from some jurisdictions for passive surveillance, maximum likelihood estimates (MLE) of prevalence were calculated in Excel (version 16.0) with 95% confidence intervals (CI) using the PooledInfRate add-in (version 4.0) ((20,21)). This estimates the probability of infection for an individual tick in the population using the results of testing of the pooled samples (i.e. a group of one or more ticks submitted and tested together). Co-infection prevalence was calculated among single submissions only to ascertain true co-infections; that is, two or more pathogens in a single tick. Where ticks were not tested in pools, prevalence was the number of positive ticks divided by the number of ticks tested.

## Results

### Passive surveillance tick characteristics

**Figure 1:**
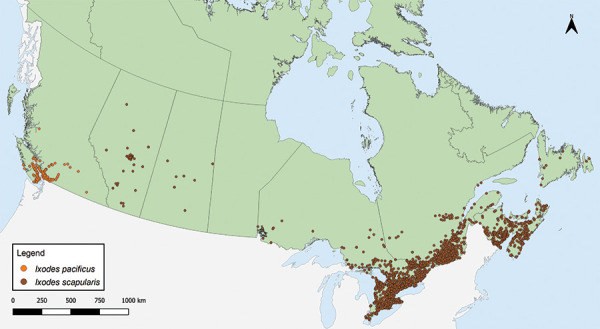
*Ixodes pacificus* and *Ixodes scapularis* ticks submitted through passive tick surveillance, Canada, 2020^a^ ^a^ Each dot represents the probable location of acquisition for an *I. pacificus* (n=718) or *I. scapularis* (n=7,397) tick submitted through passive surveillance. Ticks from Alberta Health were mapped to the centroid of the forward sortation area (first three characters of the postal code) of acquisition. One hundred and thirty-seven ticks were not mapped because the probable location of acquisition could not be determined

In 2020, a total of 8,252 ticks were submitted from nine provinces (**Table 1**, **Figure 1**). Ticks from Manitoba were excluded as five or fewer ticks were submitted. No ticks were submitted from Northwest Territories, Nunavut or Yukon. The majority (71.49%) of ticks were sample-based submissions (n=5,899) and the remainder were image-based submissions (n=2,353). Ticks from Ontario and Québec comprised 77.24% of all ticks submitted. The majority (96.80%) of ticks were from single submissions, but there were 109 multiple submissions (range: 2–6 ticks per submission; median: 2).

**Table 1:** Number of *Ixodes pacificus* and *Ixodes scapularis* ticks collected through passive surveillance by province, Canada, 2020

Province	Tick species (number of ticks)			Type of surveillance (number of ticks)^a^		Type of submission (number of submissions)^b^	
	*Ixodes pacificus*	*Ixodes scapularis*	Total	Sample-based	Image-based	Single submissions	Multiple submissions
British Columbia	718	0	718	718	N/A	670	22
Alberta	0	81	81	81	N/A	81	0
Saskatchewan	0	12	12	7	5	12	0
Ontario	0	5,139	5,139	3,713	1,426	4,964	68
Québec	0	1,235	1,235	809	426	1,208	12
Newfoundland and Labrador	0	14	14	4	10	14	0
New Brunswick	0	646	646	516	130	634	6
Nova Scotia	0	392	392	36	356	392	0
Prince Edward Island	0	15	15	15	N/A	13	1
Total	718	7,534	8,252	5,899	2,353	7,988	109

Abbreviation: N/A, not applicable

^a^ Sample-based submissions are physical tick specimens; image-based submissions are images submitted to eTick

^b^ Single submissions consist of one tick; multiple submissions consist of two or more ticks submitted together by the same individual

Tick instar, level of engorgement and host were available for 100% of *I. pacificus*. Tick instar, level of engorgement and host were available for 89.66%, 67.60% and 99.92% of *I. scapularis*, respectively. The majority of ticks submitted were adult female ticks (*I. pacificus*: 97.21%; *I. scapularis*: 92.36%) (**Table 2**). Adult males, nymphs and larvae were submitted less frequently. Overall, 8.91% of *I. pacificus* and 41.76% of *I. scapularis* were engorged. Humans were the most common host among *I. pacificus* and *I. scapularis* (90.39% and 82.98%, respectively) followed by dogs (8.91% and 13.34%, respectively).

**Table 2:** Instar, level of engorgement and host of *Ixodes pacificus* and *Ixodes scapularis* ticks submitted through passive surveillance, Canada, 2020^a^

Characteristics	Tick species			
	*Ixodes pacificus*		*Ixodes scapularis*	
	n	%	n	%
**Instar**				
Larva	0	0	9	0.13
Nymph	1	0.14	284	4.20
Adult female	698	97.21	6,239	92.36
Adult male	19	2.65	223	3.30
Total	718	100	6,755	100
**Level of engorgement**				
Engorged	64	8.91	2,127	41.76
Unfed	654	91.09	2,966	58.24
Total	718	100	5,093	100
**Host**				
Human	649	90.39	6,247	82.98
Dog	64	8.91	1,004	13.34
Cat	3	0.42	132	1.75
Other^b^	2	0.28	145	1.93
Total	718	100	7,528	100

^a^ Data are presented for all ticks where available, regardless of whether the tick was part of a single or a multiple submission

^b^ Includes environment, horse, rabbit and other unspecified animal

**Figure 2:**
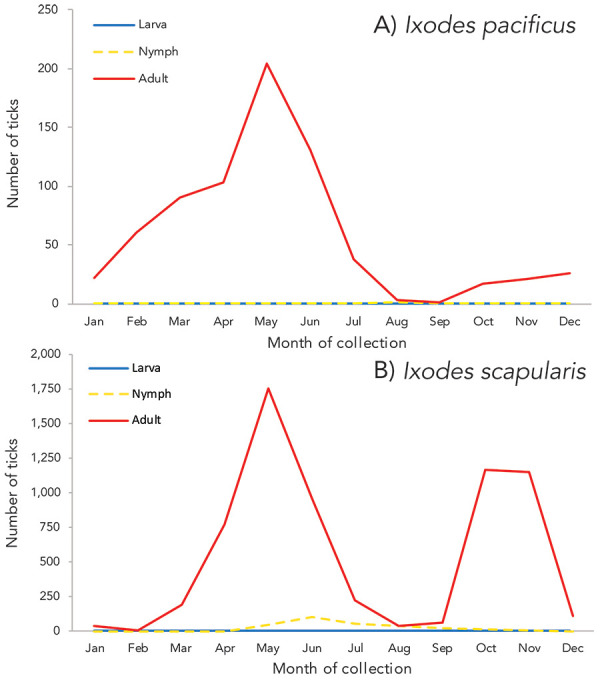
Number of *Ixodes pacificus* and *Ixodes scapularis* ticks submitted through passive surveillance, by month and tick instar, Canada, 2020^a^ ^a^ Data are presented for *I. pacificus* (n=718) and *I. scapularis* (n=6,755) ticks submitted through passive surveillance. Month of submission or tick instar was not available for *I. scapularis* (n=779)

Month of acquisition and tick instar was available for 100% of *I. pacificus* and 89.66% of *I. scapularis*. (**Figure 2**). Adult *I. scapularis* ticks submitted peaked in May and October through November, while adult *I. pacificus* submitted peaked only in May. Only 0.14% of *I. pacificus* submitted were nymphs, while 4.20% of *I. scapularis* submitted were nymphs, peaking in June. Larvae of *I. scapularis* (0.13%) were submitted June through September; no *I. pacificus* larvae were submitted.

## Passive surveillance infection prevalence

Data on laboratory testing were available for 98.27% of *I. pacificus* and 98.20%–98.40% of *I. scapularis* from sample-based submissions, depending on pathogen. The most prevalent pathogen was *B. burgdorferi*, detected in 17.19% of *I. scapularis* (95% CI: 16.17–18.26) (**Table 3**). Other tick-borne pathogens (*A. phagocytophilum*, *B. microti* and *B. miyamotoi*) and co-infections were estimated to have a prevalence rate of less than 1%. Among *I. pacificus*, only *B. miyamotoi* was identified (0.14%, 95% CI: 0.01–0.68).

**Table 3:** Prevalence of *Anaplasma phagocytophilum*, *Babesia microti*, *Borrelia burgdorferi* and *Borrelia miyamotoi* infection in *Ixodes pacificus* and *Ixodes scapularis* ticks submitted through passive surveillance, Canada, 2020^a,b^

Pathogen	Infection prevalence			
	*Ixodes pacificus*		*Ixodes scapularis*	
**Single agent**	**Maximum likelihood estimate**			
	**%**	**95% CI**	**%**	**95% CI**
*Anaplasma phagocytophilum*	N/A	N/A	0.87	0.64–1.15
*Babesia microti*	N/A	N/A	0.02	0–0.09
*Borrelia burgdorferi*	0	0–0.54	17.19	16.17–18.26
*Borrelia miyamotoi*	0.14	0.01–0.68	0.49	0.33–0.71
Total single agent	0.14	0.01–0.68	18.21	17.16–19.29
**Co-infection**	**Co-infection rate**			
	**%**	**Number co-infected ticks/number ticks tested**	**%**	**Number co-infected ticks/number ticks tested**
*Anaplasma phagocytophilum +* *Babesia microti*	N/A	N/A	0	0/4,874
*Anaplasma phagocytophilum +* *Borrelia burgdorferi*	N/A	N/A	0.12	6/4,874
*Anaplasma phagocytophilum +* *Borrelia miyamotoi*	N/A	N/A	0.02	1/4,874
*Babesia microti +* *Borrelia burgdorferi*	N/A	N/A	0	0/4,882
*Babesia microti +* *Borrelia miyamotoi*	N/A	N/A	0	0/4,883
*Borrelia burgdorferi +* *Borrelia miyamotoi*	0	0/705	0.14	7/4,882
Total co-infected	0	0/705	0.29	14/4,883

Abbreviations: CI, confidence interval; N/A, not tested

^a^ All *I. pacificus* (n=718) were not tested for *A. phagocytophilum* and *B. microti.* All *I. scapularis* from Alberta or submitted through eTick were not tested for any pathogen

^b^ Number of *I. scapularis* ticks tested: *A. phagocytophilum* (n=5,090), *B. microti* (n=5,100), *B. burgdorferi* (n=5,098), *B. miyamotoi* (n=5,094). Number of *I. pacificus* ticks tested: *B. burgdorferi* (n=705), *B. miyamotoi* (n=705)

Prevalence of *B. burgdorferi* was higher in multiple submissions of *I. scapularis* (32.31%, 95% CI: 25.27–40.34) than from single submissions (16.71%, 95% CI: 15.69–17.78). Infection prevalence did not differ significantly by submission type for any other pathogen. *Ixodes scapularis* submitted from human hosts did not have significantly different infection prevalence compared to *I. scapularis* submitted from non-human hosts.

**Figure 3:**
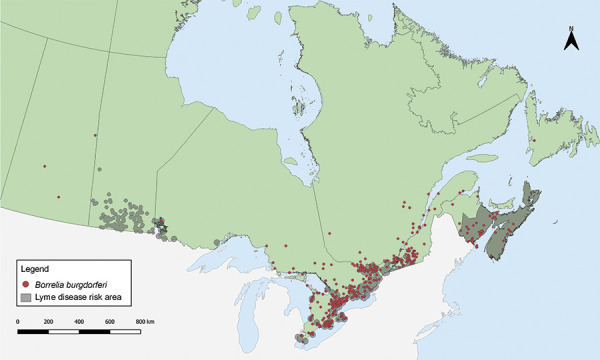
*Ixodes scapularis* ticks submitted through passive surveillance infected with *Borrelia burgdorferi*, Canada, 2020^a,b^ ^a^ Each dot represents the probable location of acquisition of at least one *I. scapularis* (n=860) single or multiple tick submission submitted through passive surveillance that was infected with *B. burgdorferi*. Eight ticks were not mapped because the probable location of acquisition could not be determined ^b^ Lyme disease risk areas are identified by the provinces as of 2021 using the methods described in the 2016 national Lyme disease case definition ((22)). On the map, risk areas are identified as hatched gray areas

**Figure 4:**
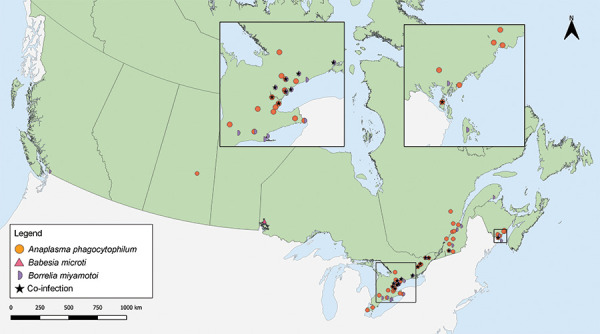
*Ixodes pacificus* and *Ixodes scapularis* ticks submitted through passive surveillance infected with *Anaplasma phagocytophilum*, *Babesia microti*, *Borrelia miyamotoi* and co-infections, Canada, 2020^a^ ^a^ Each symbol represents the probable location of acquisition of an *I. pacificus* (n=1) or *I. scapularis* (n=67) single or multiple tick submission submitted through passive surveillance that tested positive for *A. phagocytophilum* (n=42), *B. microti* (n=1), *B. miyamotoi* (n=25) or a co-infection (n=14). Co-infections were limited to only single submissions of ticks and include *B. burgdorferi* + *B. miyamotoi* (n=7), *B. burgdorferi* + *A. phagocytophilum* (n=6) and *A. phagocytophilum* + *B. miyamotoi* (n=1) all in *I. scapularis.* Two ticks with *A. phagocytophilum* and one tick with *B. miyamotoi* were not mapped because the probable location of acquisition could not be determined

Tick-borne pathogens were largely found in southern and eastern Ontario, southern Québec and southern New Brunswick (**Figure 3**, **Figure 4**, and **Table 4**). *Borrelia burgdorferi*-infected *I. scapularis* were found in six provinces: Saskatchewan, Ontario, Québec, Newfoundland and Labrador, New Brunswick and Nova Scotia. Three quarters of *B. burgdorferi*-infected *I. scapularis* submissions were within previously identified LD risk areas (74.88%; 644/860). Lyme disease risk areas are localities in which there is evidence of reproducing populations of known tick vector species (particularly *I. scapularis* and *I. pacificus*) and the likely transmission of *B. burgdorferi* ((22)). Most multiple submissions came from LD risk areas (76.15%; 83/109), of which 51.81% were infected with *B. burgdorferi* (43/83).

**Table 4:** Prevalence of *Anaplasma phagocytophilum*, *Babesia microti*, *Borrelia burgdorferi* and *Borrelia miyamotoi* infection in *Ixodes scapularis* and *Ixodes pacificus* ticks submitted through passive surveillance, by province, Canada, 2020^a^

Province	Infection prevalence Maximum likelihood estimate							
	*Anaplasma phagocytophilum*		*Babesia microti*		*Borrelia burgdorferi*		*Borrelia miyamotoi*	
	%	95% CI	%	95% CI	%	95% CI	%	95% CI
***Ixodes pacificus***								
British Columbia	N/A	N/A	N/A	N/A	0	0–0.54	0.14	0.01–0.68
***Ixodes scapularis***								
Alberta	N/A	N/A	N/A	N/A	N/A	N/A	N/A	N/A
Saskatchewan	14.29	0.85–51.51	0	0–35.43	42.86	12.96–77.51	0	0–35.43
Ontario	0.73	0.49–1.04	0.03	0–0.13	17.78	16.56–19.04	0.46	0.28–0.72
Québec	1.24	0.63–2.19	0	0–0.47	19.50	16.87–22.35	0.62	0.23–1.36
Newfoundland and Labrador	0	0–48.99	0	0–48.99	25.00	1.52–73.74	0	0–48.99
New Brunswick	1.17	0.48–2.40	0	0–0.74	8.97	6.72–11.68	0.58	0.15–1.57
Nova Scotia	0	0–9.64	0	0–9.64	25.00	13.03–40.81	0	0–9.64
Prince Edward Island	0	0–20.15	0	0–20.15	0	0–20.15	0	0–20.15
Total	0.87	0.45–1.15	0.02	0–0.09	17.19	16.17–18.26	0.49	0.33–0.71

Abbreviations: CI, confidence interval; N/A, not tested

^a^ Number of ticks tested: British Columbia (n=705), Alberta (n=0), Saskatchewan (n=7), Ontario (n=3,705–3,713), Québec (n=809), Newfoundland and Labrador (n=4), New Brunswick (n=514–516), Nova Scotia (n=36), Prince Edward Island (n=15)

*Anaplasma phagocytophilum* was found in *I. scapularis* (0.87%) in four provinces: Saskatchewan, Ontario, Québec, and New Brunswick (Figure 4, Table 4). *Borrelia miyamotoi* was found in British Columbia, Ontario, Québec and New Brunswick. *Babesia microti* was found only in Ontario. Co-infections were found in Ontario, Québec and New Brunswick.

## Active surveillance tick characteristics

In 2020, *I. scapularis* (n=688) were collected in three provinces in active surveillance: New Brunswick (n=445), Ontario (n=128) and Québec (n=115). Adult males (n=264/688; 38.37%) and females (n=214/688; 31.10%) were collected most often, followed by nymphs (n=209/688; 30.38%) and larva (1/688; 0.15%).

## Active surveillance infection prevalence

**Figure 5:**
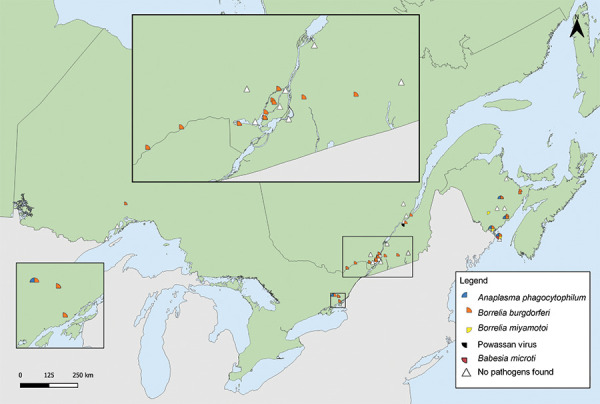
*Ixodes scapularis* ticks with associated pathogens collected through active surveillance, Canada, 2020^a,b^ ^a^ Each symbol represents an active surveillance site where *A. phagocytophilum* (n=31), *B. microti* (n=1), *B. burgdorferi* (n=200), *B. miyamotoi* (n=3), or Powassan virus (n=1) were found in *I. scapularis* ticks. There were 17 sites where no tick-borne pathogens were identified in *I. scapularis* ticks ^b^ Number of ticks tested: Ontario (n=128), Québec (n=110) and New Brunswick (n=445)

Laboratory testing results were available for 99.27% of *I. scapularis*. The most prevalent pathogen was *B. burgdorferi* (29.28%), present in Ontario, Québec and New Brunswick (**Table 5**). *Anaplasma phagocytophilum* (4.54%) was found in ticks in Ontario and New Brunswick. The remaining pathogens were found in less than 0.5% of *I. scapularis*: three *B. miyamotoi*-positive and one *B. microti*-positive ticks were found in New Brunswick, and one tick with Powassan virus (deer tick lineage) was found in Québec. The site locations where *I. scapularis* was collected in active surveillance are shown in **Figure 5**.

**Table 5:** Infection prevalence of *Ixodes scapularis* ticks collected in active surveillance, by province, Canada, 2020

Province	Infection prevalence									
	*Anaplasma phagocytophilum*		*Babesia microti*		*Borrelia burgdorferi*		*Borrelia miyamotoi*		Powassan virus	
	Proportion positive tick^a^	%	Proportion positive tick	%	Proportion positive tick	%	Proportion positive tick	%	Proportion positive tick	%
Ontario	2/128	1.56	0/128	0	53/128	41.41	0/128	0	0/128	0
Québec	0/110	0	0/110	0	40/110	36.36	0/110	0	1/110	0.91
New Brunswick	29/445	6.52	1/445	0.22	107/445	24.04	3/445	0.67	0/445	0
Total	31/683	4.54	1/683	0.15	200/683	29.28	3/683	0.44	1/683	0.15

^a^ Proportion positive tick equals the number of positive tick divided by the number of ticks tested

## Discussion

In 2020, *I. scapularis* and *I. pacificus* were submitted in passive surveillance from nine provinces. Only *I. pacificus* were submitted in British Columbia. The majority of ticks were female adults and obtained from human hosts. Among ticks that were tested, 18.21% of *I. scapularis* and 0.14% of *I. pacificus* were infected with at least one tick-borne pathogen, mainly *B. burgdorferi*. In active surveillance, five tick-borne pathogens (*A. phagocytophilum*, *B. burgdorferi*, *B. miyamotoi*, *B. microti* and Powassan virus) were identified among the *I. scapularis* collected in Ontario, Québec and New Brunswick.

From passive surveillance, 5,899 ticks were sample-based submissions, a decrease of 44% from the 10,549 ticks submitted in 2019 ((16)), which could be due, in part, to impacts from the coronavirus disease 2019 (COVID-19) pandemic. Beginning in spring 2020, COVID-19 pandemic restrictions affected traditional passive surveillance, as health units, medical clinics and veterinary clinics were limited in their ability to accept physical tick specimens at some locations (e.g. Simcoe Muskoka District Health Unit) ((23)). The decrease in submissions could also be due to changes to sample-based submission programs and greater emphasis on image-based submission programs in most jurisdictions. Active surveillance was also affected by pandemic restrictions, as in-person activities like field surveillance were limited (e.g. *Institut national de santé publique du Québec*) ((24)). Data from the Canadian Lyme Sentinel Network, which was included in the 2019 report ((16)), was unavailable in 2020 as Canadian Lyme Sentinel Network activities were suspended (*personal communication, C. Guillot, 2022*).

In passive surveillance, ticks were submitted every month, but submissions followed distinct species-specific patterns influenced by location and weather. Despite fewer ticks submitted to passive surveillance than in 2019 ((16)), the same bimodal peaks for *I. scapularis* adults that have been shown historically in central and eastern Canada ((13,25–27)) were observed in 2020. For *I. pacificus*, a single springtime peak was observed as shown previously in British Columbia ((14,16)) and the western United States ((28)). While risk of exposure to ticks was present year-round, exposure to tick-borne pathogens is dependent on infection prevalence and attachment time.

The proportion of ticks submitted from dogs or cats increased from 8.9% in 2019 to 15.1% in 2020 ((16)). This increase is likely from including data from eTick: whereas sample-based passive surveillance programs in some localities (e.g. health units, municipalities) are restricted to ticks from human hosts only, image-based passive surveillance has no such restriction, leading to a greater proportion of ticks from animal hosts when eTick data was included in this report.

Compared to 2019 ((16)), province and pathogen-specific infection prevalence estimates were similar, but geographic distribution was more limited in some cases (e.g. *I. scapularis* with *A. phagocytophilum* were limited to only the southernmost parts of New Brunswick compared to 2019). Several factors influence infection prevalence estimates from year-to-year or between provinces, including annual variation in weather, surveillance effort, habitat suitability, presence of established vector and reservoir populations and interactions between humans, ticks and the environment. Because of small sample sizes tested (n=<10), infection prevalence estimates from Saskatchewan and Newfoundland and Labrador should be interpreted with caution.

*Ixodes pacificus* (found in British Columbia) historically have low rates of *B. burgdorferi* infection ((14,16)), while *B. burgdorferi* infection prevalence in *I. scapularis* found in central and eastern Canada is typically higher ((18,25,29)); both trends continued to be observed in 2020. Jacob *et al.* ((30)) report higher infection prevalence among companion animals of several tick-borne pathogens compared to our estimates; however, participating veterinary clinics in that study were skewed towards areas with higher or emerging risk of TBD, likely leading to overestimation of the province-level infection prevalence. The one-year study also concluded in spring 2020, thus not accounting for the effects of pandemic restrictions on tick exposure for the remainder of 2020.

The majority of *B. burgdorferi*-infected *I. scapularis* had probable location of acquisition within LD risk areas ((8,22)). The remaining *B. burgdorferi*-infected *I. scapularis* may be adventitious ticks carried by migrating birds or mammals ((15)) or collected from areas with emerging LD risk. Provinces routinely review LD risk areas based on new surveillance data according to the 2016 case definition ((22)).

Despite limited opportunities for active field surveillance due to ongoing COVID-19 pandemic restrictions, over 600 *I. scapularis* were collected in drag sampling from 45 sites across Ontario, Québec and New Brunswick. Five tick-borne pathogens were identified, ranging in prevalence from 0.15% to 29.28%. This was the first detection of Powassan virus (deer tick lineage) in active surveillance in Québec ((24)), which has previously been identified in small numbers of *Ixodes* spp. in Manitoba, Ontario and New Brunswick ((12,31)).

In addition to single-agent infection with *B. burgdorferi* and the four other tick-borne pathogens, three distinct types of co-infections were identified. Surveillance beyond LD for other TBD is warranted to monitor the emergence and spread of these pathogens, especially as suitable habitat for *Ixodes* spp. is predicted to increase due to changes in climate and environment ((1,32,33)).

Co-infections have been reported to varying extents in ticks found in Canada ((16,18)) and the United States ((34)). Humans who are co-infected may experience a greater number and duration of symptoms compared to single-agent infections ((35,36)). Many factors influence the risk of co-infection, including attachment time, but preventing tick bites can help prevent transmission of all TBDs.

## Strengths and limitations

This article presents a snapshot of infection prevalence and range estimates for the main LD vectors in Canada. While traditional passive surveillance programs have been discontinued or limited to specific hosts in some regions, incorporating data from eTick allows broader geographic and host representation from these regions in this summary. Combining passive and active surveillance also allows the strengths and weaknesses of the systems to complement each other. For example, while active surveillance is limited in geographic and temporal scope, passive surveillance programs gather data from large areas throughout the year.

There are several limitations to this study. Due to competing public health priorities, passive surveillance programs and the effort of active surveillance vary across Canada. As previously noted, COVID-19 pandemic restrictions affected public health services and surveillance in 2020, resulting in fewer sample-based submissions to passive surveillance and active surveillance that was less geographically representative compared to the previous year ((16)). Shifts in passive tick surveillance programs (e.g. limits on tick host or location of acquisition of tick; discontinuation of regional or provincial programs) have also limited the number of submissions. While digital platforms like eTick offer timely tick identification, tick specimens are not routinely requested for tick-borne pathogen testing from imaging identification platforms ((17)). Recall bias in reporting locality of acquisition and travel history in passive surveillance might create uncertainty as to the exact location where ticks were found. Finally, there are likely other active surveillance programs conducted in 2020 not included here in this summary if ticks were not sent for pathogen testing at NML. Furthermore, the number of larvae included in active surveillance is an underestimate, since our dataset only includes ticks sent for testing, for which larvae are rarely sent. These underestimates of the number of ticks may affect the accuracy of infection prevalence of various pathogens.

## Conclusion

*Ixodes scapularis* and *I. pacificus* were identified across Canada in passive and active surveillance, some of which were infected with *B. burgdorferi*, the LD pathogen, but also with emerging tick-borne pathogen(s). Healthcare professionals and the public should be aware that there is a risk of exposure to infected ticks outside of known LD risk areas, even if the risk is low in those areas. The identification of new tick-borne pathogens in several jurisdictions in active surveillance may help public health authorities update their prevention strategies, as some of those emerging tick-borne illnesses, like Powassan virus disease, may have infection transmission patterns that differ from LD. As climate change alters the habitat and seasonality of tick vectors, continued surveillance can help in timely identification of new risk areas for LD and other emerging TBD, and directing public health interventions towards these at-risk areas.
